# Commentary: Tissue eosinophil level as a predictor of control, severity, and recurrence of Chronic Rhinosinusitis with Nasal Polyps

**DOI:** 10.3389/falgy.2025.1628864

**Published:** 2025-06-26

**Authors:** Matteo Gelardi

**Affiliations:** Department of Otolaryngology, University of Foggia, Foggia, Italy

**Keywords:** CRSwNP recurrent, mast-cell, nasal cytology, Clinical-Citological Grading, eosinophils, microscopy

A Commentary on Tissue eosinophil level as a predictor of control, severity, and recurrence of Chronic Rhinosinusitis with Nasal Polyps By Vizcarra-Melgar J, Sánchez-Gómez S, López-González N, Moreno-Luna R, González-García J and Maza-Solano J (2025). Front. Allergy 6:1549332. doi: 10.3389/falgy.2025.1549332

Dear Editor,

I read with great interest the paper by Vizcarra-Melgar and colleagues, which revisits the belief that tissue eosinophil counts alone can stratify chronic rhinosinusitis with nasal polyps (CRSwNP). Their large series and use of POLINA composite criteria are commendable. Yet several methodological and conceptual issues, detailed below, suggest that the negative results may derive more from study design than from genuine irrelevance of tissue data (1).

## 1 A pronounced ceiling effect

In the cohort, 81.5% of specimens exceeded the ≥10 eosinophils·HPF-^1^ threshold and more than half surpassed 55 eosinophils·HPF-^1^ (1). When a predictor clusters near its upper bound, statistical power to discriminate outcomes collapses. Re-analysing the dataset with higher cut-offs or, preferably, modelling eosinophils as a continuous variable (e.g., restricted cubic splines) could unmask hidden dose–response trends.

## 2 The added value of the Clinical-Cytological Grading (CCG)

Nasal cytology—simple, rapid, inexpensive—captures inflammatory endotypes that routine histology misses (2). The Clinical-Cytological Grading (CCG) combines the dominant cellular pattern (eosinophils, mast cells, neutrophils, or mixed) with “type-2 amplifiers” such as asthma, aspirin-exacerbated respiratory disease and inhalant allergy. A score ≥7 correlates with poor disease control and higher Lund–Mackay scores (3, 4), and underpins the Prognostic Index of Recurrence (PIR) prospectively linked to post-surgical relapse (3). By integrating local and systemic information, CCG avoids the tunnel vision of single-biomarker approaches. Stratifying the present dataset by CCG might show that histological eosinophilia seems irrelevant only because the inflammatory milieu was incompletely captured.

## 3 The missing mast-cell piece

Histopathology was labelled eosinophilic, neutrophilic or mixed, but mast cells were not quantified. This omission matters. Intra-epithelial mast cells co-localise with eosinophils in recalcitrant CRSwNP and parallel severe anosmia (4); their presence independently predicts early relapse after functional endoscopic sinus surgery (5). IgE-bearing mast cells are plausible drivers of “type 2-high” remodelling and of the favourable response to omalizumab. Neglecting them likely underestimates local inflammatory load and helps explain the weak linkage between tissue data and clinical endpoints.

## 4 Harmonising outcome definitions

The authors sensibly adopted POLINA to grade severity and control. Earlier histopathological series, however, relied on SNOT-22, VAS or Lund–Mackay alone. Because these indices capture overlapping yet distinct domains, comparing effect sizes across studies is problematic. A consensus framework that cross-walks CCG, POLINA, EPOS control criteria and biological “passport” variables (blood eosinophils, total IgE, periostin) would enhance reproducibility and enable meta-analytic synthesis.

## 5 Practical implications

Clinicians favour tools that are quick, cheap and predictive. A May-Grünwald-Giemsa smear takes under ten minutes, costs pennies and has a gentle learning curve (2). One slide reveals the endotype, flags a high CCG (≥7) with elevated relapse risk, and guides decisions on biologics or peri-operative oral steroids (6). Until validated molecular panels become affordable, CCG offers a pragmatic bridge between histology and bench-top omics.

## 6 Suggestions for future research

• Apply continuous or higher eosinophil thresholds to blunt the ceiling effect.
• Quantify mast cells systematically (May-Grünwald-Giemsa or tryptase immunohistochemistry) to refine tissue phenotypes (7).
• Compare CCG, POLINA severity and blood biomarkers head-to-head in prospective multicentre designs.
• Validate the PIR in non-Mediterranean cohorts to test geographic generalisability.
• Integrate multi-omics (transcriptome, proteome) with cytology to uncover targets beyond the IL-4/IL-5/IgE axis (8).

## 7 Conclusion

Vizcarra-Melgar et al. convincingly show that low-threshold tissue eosinophilia alone cannot explain the full clinical spectrum of CRSwNP (1). Their data illustrate the limits of single-cell-type metrics rather than the futility of tissue analysis. Incorporating the Clinical-Cytological Grading and explicitly counting mast cells can overcome the ceiling effect, capture mixed inflammatory phenotypes and yield a pragmatic bedside prognostic index—aligning with precision-medicine ambitions and benefiting patients with this stubbornly relapsing disease.
